# Anterior cruciate ligament reconstruction with six and eight‐strand hamstring tendon autografts produces adequate graft dimensions and functional outcomes: A systematic review

**DOI:** 10.1002/ksa.12556

**Published:** 2024-12-12

**Authors:** Bryan Sun, Boss Lee, Justin Grad, Dan Cohen, Jihad Abouali, Sachin Tapasvi, Adit Maniar, Darren de SA

**Affiliations:** ^1^ Michael G. DeGroote School of Medicine, Faculty of Health Sciences McMaster University Hamilton Ontario Canada; ^2^ Department of Surgery, Division of Orthopaedic Surgery McMaster University Hamilton Ontario Canada; ^3^ Division of Orthopaedic Surgery Michael Garron Hospital Toronto Ontario Canada; ^4^ The Orthopaedic Specialty Clinic Pune Maharashtra India; ^5^ Department of Orthopaedics London Health Sciences Centre London Ontario Canada

**Keywords:** ACL graft, anterior cruciate ligament, hamstring, review of ACL reconstruction

## Abstract

**Purpose:**

This study aims to summarize the graft dimensions, failure rates, return‐to‐sport rates and patient‐reported outcome measures (PROMs) following anterior cruciate ligament reconstruction (ACLR) with six or eight‐strand hamstring tendon autografts (6SHG or 8SHG).

**Methods:**

Three databases were searched from inception to 12 February 2024. The authors adhered to PRISMA guidelines and the Cochrane Handbook for Systematic Reviews of Interventions. All clinical studies reporting patient demographics, objective clinical outcomes and PROMs following ACLR with 6SHG or 8SHG were included for data synthesis. PROMs included the International Knee Documentation Committee (IKDC), Lysholm and Tegner scores.

**Results:**

Thirteen studies comprising 1103 patients were included (mean age: 30.6 years). The transtibial technique was used in all studies, except one study using anatomic ACLR (*n* = 38), and one study using transtibial and all‐inside ACLR (*n* = 41). Eight studies comprising 512 patients used 6SHG, four studies comprising 507 patients 8SHG and two studies comprising 97 patients used either. Mean graft diameters ranged from 8.0 to 9.2 mm (6SHG) and 9.1 to 9.9 mm (8SHG). Mean graft lengths for 49 6SHG patients ranged from 60.0 to 83.3 mm. The failure rate for 817 patients (6SHG or 8SHG) was 4.8% (0.0%–20.0%). The return‐to‐sport rate for 112 patients (6SHG or 8SHG) was 75.9% (69.7%–100.0%). Mean IKDC, Lysholm and Tegner scores for 6SHG or 8SHG were 88.4 (86.1–96.3), 91.7 (90.4–96.5) and 6.9 (6.5–7.3), respectively.

**Conclusions:**

Both 6SHG and 8SHG produced graft diameters <8 mm. Data regarding PROMs suggested good patient satisfaction based on established criteria. Re‐rupture and return‐to‐sport rates were 4.8% and 75.9%, respectively.

**Level of Evidence:**

Level IV.

**Abbreviations**
4SHG: four‐strand hamstring tendon autograft
6SHG: six‐strand hamstring tendon autograft
8SHG: eight‐strand hamstring tendon autograft
ACLR: anterior cruciate ligament reconstruction
BPTB: bone‐patellar tendon‐bone
IKDC: International Knee Documentation Committee
MINORS: methodological index for nonrandomized studies
PRISMA: preferred reporting items for systematic reviews and meta‐analyses
PROM: patient‐reported outcome measure
RCT: randomized controlled trial

## INTRODUCTION

Each year, nearly 250,000 anterior cruciate ligament (ACL) injuries occur in Canada and the United States, with the incidence rising due to increasing sports participation [5, 18]. Both operative and nonoperative treatments are options for ACL tears; however, nonoperative management has been associated with significantly higher rates of knee instability and lower rates of return to sport [42]. As a result, ACL reconstruction (ACLR) is routinely recommended for active individuals, especially younger patients, aiming to return to preinjury activity levels and avoid activity limitations [32].

Among autograft options to replace the physiologic ACL, bone‐patellar tendon‐bone (BPTB) and hamstring grafts are the most commonly used [35]. A survey conducted in May 2020 of over 2000 orthopaedic surgeons found that 80.3% preferred hamstring autografts for primary reconstructions [43]. Compared to BPTB autografts, hamstring autografts offer advantages such as reduced donor site morbidity, decreased anterior knee pain and smaller incisions. However, hamstring autografts are associated with slightly higher rates of graft failure and unpredictable final graft diameters [27, 36]. Reliable graft thickness is crucial in ACLR since autografts with diameters under 8.0 mm are correlated with reduced tensile strength and higher rates of ACL revision surgery [38]. Accordingly, multiple studies have recommended hamstring autografts to carry a minimum diameter of 8.0 mm [15].

Historically, four‐strand hamstring tendon autografts (4SHG) have been the most common preparation method for hamstring autograft; however, the average intraoperative diameter is frequently <8 mm, with some studies even noting it to be <7 mm [7]. Factors such as a patient's height, sex and mass of the patient have all been correlated with final thickness, with skeletally immature patients and those of a smaller stature at greater risk of having an insufficient 4SHG [44]. While recent studies have shown preoperative magnetic resonance imaging to be moderately correlated with intraoperative graft diameter, it cannot guarantee an adequate final graft size [1].

Increasing the number of strands in the autograft from the standard 4SHG to 6SHG or 8SHG has recently been explored as a method to conveniently and reliably enlarge the final graft diameter. While numerous factors determine postoperative success, it is hypothesized that using greater stranded grafts will ensure sufficient graft diameters, ultimately improving graft reliability and patient‐reported outcome measures (PROMs) [31]. One possible drawback is that increasing the number of strands may result in a graft length that is mismatched with the ideal bone tunnel length [46]. This systematic review aims to summarize the graft dimensions, objective clinical outcomes and PROMs associated with 6SHG and 8SHG.

## MATERIALS AND METHODS

The Preferred Reporting Items for Systematic Reviews and Meta‐Analyses and Revised Assessment of Multiple Systematic Reviews guidelines for coordinating and reporting systematic reviews were followed during the development of this research [20, 22].

### Search criteria

Three online databases (PubMed, MEDLINE and EMBASE) were searched on 12 February 2024 to evaluate the use of 6SHG and 8SHG in ACLR. Comprehensive search terms including ‘anterior cruciate ligament’, ‘ACL’, ‘hamstring’ and ‘strand’ were utilized (Supporting Information S1: Table 1).

Studies were selected for inclusion if they met the following criteria: (1) studies with patients undergoing ACLR (no age or physeal status restriction); (2) studies with a specific patient group or subgroup receiving 6SHG or 8SHG; (3) studies reporting graft dimensions, objective outcomes (i.e., graft failure, return to sport, etc.) or PROMs; (4) human studies and (5) studies published in the English language. Exclusion criteria included the following: (1) revision ACLR or multiligament knee reconstructions; (2) level of evidence V; (3) textbook chapters, systematic reviews or meta‐analyses; (4) conference abstracts and (5) biomechanical, cadaveric or animal studies. There was no minimum follow‐up period required for inclusion. The reference sections of included studies were also assessed for possible eligibility.

### Screening

Title and abstract screening was conducted independently and blindly by two authors (B. S. and B. L.), with conflicts resolved through consensus or consultation with a more senior author (D. D. S.) if no consensus was reached. During the full‐text screening stage, studies were independently screened by the initial two authors, and disagreements were resolved similarly.

### Assessment of agreement

The interreviewer agreement was evaluated using the kappa (*κ*) statistic for screening. A priori classification was determined using the following criteria: *κ* of 0.91–0.99 was considered to be almost perfect agreement; κ of 0.71–0.90 was considered to be considerable agreement; *κ* of 0.61–0.70 was considered to be high agreement; *κ* of 0.41–0.60 was considered to be moderate agreement; *κ* of 0.21–0.40 was considered to be fair agreement and *κ* of 0.20 or less was considered to be no agreement [26].

### Quality assessment

Quality assessment was conducted independently by two authors (B. S. and J. G.). The methodological index for nonrandomized studies (MINORS) criteria was used for the quality assessment of nonrandomized studies [37]. Based on the MINORS criteria, noncomparative and comparative studies could get a maximum score of 16 and 24, respectively [37]. For noncomparative studies, scores of 0–4 indicated very low‐quality evidence, 5–7 indicated low‐quality evidence, 8–12 indicated fair quality and ≥13 indicated high‐quality evidence. For comparative studies, scores of 0–6 indicated very low‐quality evidence, 7–10 indicated low‐quality evidence, 11–15 indicated fair‐quality evidence, 16–20 indicated good‐quality evidence and ≥20 indicated high quality [8]. The Detsky Quality Assessment Scale was used for the quality assessment of randomized controlled trials (RCT) [9]. The scale consists of 14 questions categorized into (1) randomization, (2) outcome measures, (3) inclusion and exclusion criteria and reasons for patient exclusion, (4) interventions and (5) statistical analysis. All five categories are weighted equally, each obtaining a maximum of four points. The statistical analysis category contains an additional question specific to RCTs with negative findings (e.g., if a trial was negative, were confidence intervals or post hoc calculations performed?). Therefore, the maximum score for RCTs with positive findings was 21, and RCTs with negative findings was 22 [9].

### Data abstraction and reporting

Two authors (B. L. and J. G.) independently extracted and summarized data from included articles using Google Sheets software (Google LLC). Demographic data such as number of patients, follow‐up time, sex and mean age were recorded. Outcomes included graft diameter, graft length, PROMs, rates of positive Lachman test, positive pivot shift test and graft rupture. If present, data on concomitant procedures (e.g., meniscal allograft transplantation, cartilage surgery, etc.) were also recorded. Results were presented in a descriptive summary format using Google Sheets (Google LLC). Pooled means with associated standard deviations or ranges were calculated for continuous outcomes. Frequencies and associated percentages were calculated for dichotomous outcomes. If a study performed a subgroup analysis with associated statistical parameters, *p* values were recorded. A *p *< 0.05 was considered statistically significant.

## RESULTS

### Literature search

**Figure 1:**
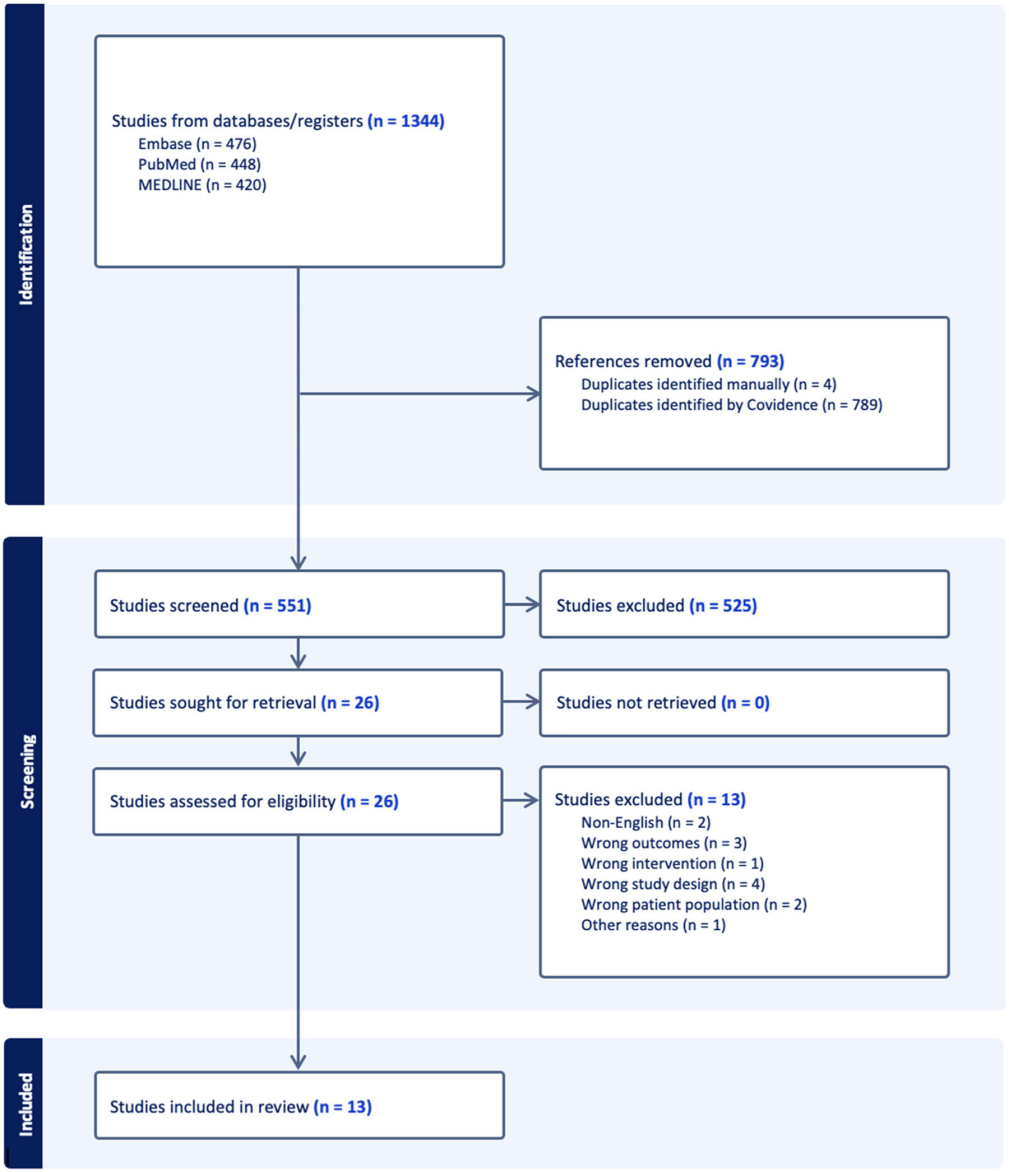
Preferred Reporting Items for Systematic Reviews and Meta‐Analyses flow diagram representing a systematic review summarizing the graft dimensions, failure rates, return to sport rates, patient‐reported outcome measures and other related measures following anterior cruciate ligament reconstruction with six or eight‐strand hamstring tendon autografts.

The initial search of the databases yielded 1344 studies, with 793 being automatically filtered out as duplicates. Following title and abstract screening, 526 studies were deemed irrelevant, leaving 25 studies for full‐text screening. Ultimately, 13 full‐text articles satisfied our inclusion and exclusion criteria (Figure 1) [2, 4, 10, 12, 21, 23, 30, 33, 40, 45, 48, 49, 50]. There was considerable agreement during the title and abstract (*k* = 0.884) stage and near‐perfect agreement during the full‐text (*k* = 0.920) stage.

### Study quality

The 13 studies in this review included two case series (Level IV evidence) [2, 50], five retrospective cohort studies (Level III evidence) [4, 10, 33, 40, 48], one prospective cohort study (Level III evidence) [30], one case‐control study (Level III evidence), one lesser quality RCT (Level II evidence) [49] and three higher‐quality RCTs (Level I evidence) [12, 21, 23]. The mean MINORS score was 10.5 for noncomparative studies and 18.4 for comparative studies. Four studies were of fair quality, and five were of good quality. The mean Detsky score for RCTs was 16.0/21 (Table 1).

**Table 1:** Demographics.

Author (year)	Study design (level of evidence)	Number of patients	Follow‐up time ± SD (months)	Female (%)	Mean age ± SD (years)	Loss to follow‐up (%)	MINORS/Detsky
El‐Azab (2023)	Randomized controlled trial (I)	48	Minimum: 24.0	14.6	27.0 ± 5.9	4.0	15/21 (Detsky)
Ye (2023)	Retrospective cohort study (III)	410	99.7 ± 26.4	26.1	26.8 ± 8.4	NR	20/24 (MINORS)
Dietvorst (2023)	Retrospective cohort study (III)	17	NR	72.0	16.0 (range: 15.8–17.0)	NR	18/24 (MINORS)
Alkhalaf (2021)	Case series (IV)	41	Minimum: 24.0	NR	NR	NR	10/16 (MINORS)
Nazari (2021)	Prospective cohort study (III)	78	NR	0.0	78.0 ± 5.9	NR	16/24 (MINORS)
Attia (2020)	Retrospective cohort study (III)	156	47.8 (range: 46.1–49.5)	2.6	32.4 **±** (range: 31.3–33.3)	NR	19/24 (MINORS)
Perkins (2019)	Retrospective cohort study (III)	65	26.0 (range: 6.0–56.0)	72.0	16.0 ± 1.5	6.0	19/24 (MINORS)
Tutkus (2018)	Case‐control study (III)	74	NR	28.4	30.5 ± 8.7	NR	12/16 (MINORS)
Liu (2016)	Randomized controlled trial (I)	80	80.4	17.4	27.7	15.0	20/21 (Detsky)
Sun (2013)	Retrospective cohort study (III)	32	34.5 (range: 26.0–39.0)	71.9	28.0 (range: 23.0–36.0)	NR	12/16 (MINORS)
Laoruengthana (2009)	Randomized controlled trial (I)	17	18.9 (range: 12.0–31.0)	5.9	28.7	NR	18/21 (Detsky)
Zhao (2007)	Lesser‐quality randomized controlled trial (II)	38	38.0 (range: 24.0–48.0)	39.5	24.0 (range: 16.0–39.0)	7.9	11/21 (Detsky)
Zhao (2006)	Case series (IV)	47	Minimum: 12.0	44.2	22.0	NR	8/16 (MINORS)

Abbreviations: MINORS, methodological index for nonrandomized studies; NR, not reported; SD, standard deviation.

### Study characteristics

Of the 13 included studies, there were 1103 patients (1103 knees), with an average of 84.8 patients per study [2, 4, 10, 12, 21, 23, 30, 33, 40, 45, 48, 49, 50]. The mean follow‐up time of seven studies that reported values was 49.3 months (range: 18.9−99.7) [4, 21, 23, 33, 40, 48, 49], and the mean loss to follow‐up of four studies that reported values was 9.0% [12, 23, 33, 49]. Twelve studies reported on patient sex with females comprising 25.6% of patients [4, 10, 12, 21, 23, 30, 33, 40, 45, 48, 49, 50]. Twelve studies comprising 1062 patients reported age values with the weighted mean being 30.6 years (range: 16.0−78.0) [4, 10, 12, 21, 23, 30, 33, 40, 45, 48, 49, 50]; two of these studies comprising 82 patients assessed paediatric or adolescent patients (defined as age ≤18 years) with a weighted mean age of 16.0 years [10, 33]. The transtibial ACLR technique was used in all studies except for one study comprising 38 patients that used anatomic ACLR [49], and another study comprising 41 patients that used both transtibial and all‐inside ACLR [2]. Eight studies comprising 512 patients evaluated the outcomes of 6SHG [2, 4, 12, 21, 30, 33, 40, 45]; 424 patients (82.8%) received a tripled semitendinosus and gracilis autograft (3ST + 3GT), 65 patients (12.7%) received a doubled semitendinosus and gracilis autograft with allograft augmentation (6STGAllo) and 23 patients (4.5%) received a quadrupled semitendinosus and doubled gracilis autograft (4ST + 2GT). Four studies comprising 507 patients evaluated the outcomes of 8SHG with all receiving a quadrupled semitendinosus and gracilis construct (4ST + 4GT) [2, 48, 49, 50]. An additional two studies comprising 97 patients did not specify whether patients received 6SHG or 8SHG and evaluated both collectively [10, 23]. Details on graft constructs are outlined in Table 2.

**Table 2:** Graft details and dimensions.

Author	Graft construct	Graft diameter ± SD (mm)	Graft length (mm)
El‐Azab (2023)	6SHG: 3ST + 3GT	9.5 ± 2.5	NR
Ye (2023)	8SHG: 4ST + 4GT	9.9 ± 0.7	NR
Dietvorst (2023)	6SHG: 3ST + 3GT (*n* = 13) 8SHG: 4ST + 4GT (*n* = 4)	8.5 (range: 8.0–9.0)	NR
Alkhalaf (2021)	6SHG: 3ST + 3GT (*n* = 29) 8SHG: 4ST + 4GT (*n* = 12)	8.6 ± 0.7 9.1 ± 0.7	NR
Nazari (2021)	6SHG: 3ST + 3GT	9.2 ± 0.7	NR
Attia (2020)	6SHG: 3ST + 3GT	9.0 (range: 8.9–9.0)	Minimum: 80.0
Perkins (2019)	6SHG: 6STGAllo	9.2 ± 0.5	NR
Tutkus (2018)	6SHG: 3ST + 3GT	Femoral side: 9.1 Tibial side: 9.7	Minimum: 90.0
Liu (2016)	6SHG: 3ST + 3GT 8SHG: 4ST + 4GT	NR	Minimum anteromedial bundle: 70.0 Minimum posterolateral bundle: 65.0
Sun (2013)	6SHG: 3ST + 3GT (*n* = 9) 6SHG: 4ST + 2GT (*n* = 23)	7.5 ± 0.4 8.0 ± 0.6	60.0
Laoruengthana (2009)	6SHG: 3ST + 3GT	9.5 (range: 8.0–11.0)	83.3 (range: 78.0–86.0)
Zhao (2007)	8SHG: 4ST + 4GT	NR	NR
Zhao (2006)	8SHG: 4ST + 4GT	NR	NR

Abbreviations: 3ST + 3GT, tripled semitendinosus and gracilis autograft; 4ST + 2GT, quadrupled semitendinosus and doubled gracilis autograft; 6SHG, six‐strand hamstring tendon autograft; 6STGAllo, doubled semitendinosus and gracilis autograft with allograft augmentation; 8SHG, eight‐strand hamstring tendon autograft; NR, not reported; SD, standard deviation.

### Graft diameter

Eight studies comprising 512 patients receiving 6SHG reported on mean graft diameters [2, 4, 12, 21, 30, 33, 40, 45]. Four hundred and twenty‐four patients receiving 3ST + 3GT constructs reported mean graft diameters ranging from 7.5 to 9.5 mm, 65 patients receiving 6STGAllo constructs reported a mean graft diameter of 9.2 mm and 23 patients receiving 4ST + 2GT constructs reported a mean graft diameter of 8.0 mm. Two studies comprising 422 patients receiving 8SHG (4ST + 4 GT) reported on mean graft diameters, with values ranging from 9.1 to 9.9 mm [2, 48]. An additional study comprising 17 patients receiving 6SHG or 8SHG reported a mean graft diameter of 8.5 mm [10]. Details on graft diameter are summarized in Table 2.

### Graft length

Four studies comprising 279 patients receiving 6SHG (3ST + 3GT or 4ST + 2GT) reported on graft lengths. Two of these studies reported mean graft lengths ranging from 60.0 to 83.3 mm [21, 40], while the other two reported minimum graft lengths ranging from 80.0 to 90.0 mm [4, 45]. An additional study comprising 40 patients receiving double‐bundle 6SHG or double‐bundle 8SHG reported minimum graft lengths of 70.0 and 65.0 mm for the anteromedial and posterolateral bundles, respectively [23]. Details on graft length are summarized in Table 2.

### Objective clinical measures

Seven studies comprising 835 patients reported graft failure rates, defined as persistent or recurrent instability and/or revision ACLR [2, 4, 12, 21, 23, 33, 48]. Of these, 286 6SHG patients with a mean follow‐up of 39.8 months had a failure rate of 7.1% (range of means: 0.0%–20.0%), while 410 8SHG patients with a follow‐up of 99.7 months had a failure rate of 3.9%. The overall failure rate of 817 6SHG or 8SHG patients with a mean follow‐up of 78.0 months was 4.8% (range of means: 0.0%–20.0%). Four studies comprising 189 patients with a mean follow‐up of 67.3 months reported on postoperative Lachman testing, with seven knees (3.7%) being grade I and one knee (0.5%) being grade II [12, 23, 40, 50]. Six studies comprising 237 patients with a mean follow‐up of 55.7 months reported on postoperative pivot shift testing with 33 knees (13.9%) being grade 1, three knees (1.3%) being grade 2 and three knees (1.3%) being grade 3 [12, 21, 33, 40, 49, 50].

Five studies comprising 522 patients reported return to sport rates [12, 21, 23, 40, 48]. Of these, 46 6SHG patients with a mean follow‐up of 36.4 months had an overall return to sport rate of 84.8% (range of means: 84.4%–100.0%), while 410 8SHG patients with a follow‐up of 99.7 months had a return to preinjury level of sports rate of 37.3%. The overall return to sport rate of 112 6SHG or 8SHG patients with a mean follow‐up of 90.5 months was 75.9% (range of means: 69.7%–100.0%). Details on objective clinical measures are summarized in Table 3.

**Table 3:** Clinical outcomes.

Author	Objective outcome measures					Subjective outcome measures		
	Graft failure (%)	Return to sport (%)	Return to preinjury sports level (%)	Positive Lachman	Positive pivot shift	IKDC ± SD	Lysholm ± SD	Tegner ± SD
El‐Azab (2023)	6.3	100.0	NR	0/48	12/48 (8 grade I, 3 grade II, 1 grade III)	87.9	NR	NR
Ye (2023)	3.9	NR	37.3	NR	NR	86.1 ± 11.0	90.4 ( ± 9.5)	6 (median)
Alkhalaf (2021)	2.1	NR	NR	NR	NR	NR	NR	NR
Attia (2020)	2.7	NR	NR	NR	NR	NR	NR	NR
Perkins (2019)	20.0	NR	NR	NR	NR	NR	NR	NR
Liu (2016)	2.9	69.7	NR	0/66	21/66 (19 grade I, 2 grade III)	92.8	95.6	7.3
Sun (2013)	NR	84.4	NR	6/32 (grade I)	1/32 (grade I)	NR	92 (median)	4 (median)
Laoruengthana (2009)	0.0	84.6	NR	NR	2/13 (grade I)	90.9	92.0	NR
Zhao (2007)	NR	NR	NR	NR	1/35 (grade I)	96.3 ± 2.8	96.5 ± 2.9	6.7 ± 0.8
Zhao (2006)	NR	NR	NR	2/32 (1 grade I, 2 grade II)	2/43 (grade I)	95.7 ± 3.1	94.1 ( ± 2.5)	6.4 ± 0.6

Abbreviations: IKDC, International Knee Documentation Committee; NR, not reported; SD, standard deviation.

### Subjective clinical measures

PROMs included in this review were the International Knee Documentation Committee (IKDC), Lysholm and Tegner scores [17]. Six studies comprising 626 6SHG or 8SHG patients with a mean follow‐up of 90.0 months had a pooled mean IKDC score of 88.4 (range of means: 86.1–96.3) [12, 21, 23, 48, 49, 50]. In particular, 65 6SHG patients with a mean follow‐up of 69.6 months had mean IKDC scores ranging from 87.9 to 90.9, while 8SHG 495 patients with a mean follow‐up of 94.5 months had mean IKDC scores ranging from 86.1 to 96.3. One study comprising 48 patients receiving 6SHG reported a correlation between younger age and higher IKDC scores (*r* = −0.825, *p* < 0.001) [12], while another study comprising 38 patients reported higher IKDC scores with 8SHG compared to 4HS (96.3 vs. 86.4, *p* = 0.007) [49].

Five studies comprising 578 6SHG or 8SHG patients with a mean follow‐up of 90.0 months had a pooled mean Lysholm score of 91.7 (range of means: 90.4–96.5) [21, 23, 48, 49, 50]. Of these, 17 6SHG patients with a follow‐up of 18.9 months reported a mean Lysholm score of 92.0, while 495 8SHG patients with a mean follow‐up of 94.5 months reported mean Lysholm scores ranging from 90.4 to 96.5. Another study comprising 32 6SHG patients with a follow‐up of 34.5 months receiving 6SHG reported a median Lysholm score of 92 [40]. One study comprising 38 patients receiving 8SHG reported higher Lysholm scores than those receiving 4HS (96.5 vs. 89.6, *p* < 0.001) [49].

Three studies comprising 151 6SHG or 8SHG patients with a mean follow‐up of 66.7 months had a pooled mean Tegner score of 6.9 (range of means: 6.5–7.3) [23, 49, 50]. Two additional studies, each comprising 32 6SHG and 410 8SHG patients, reported median Tegner scores of 4 and 6, respectively [40, 48]. Similar to IKDC and Lysholm, one study comprising 38 patients receiving 8SHG reported higher mean Tegner scores than those receiving 4HS (6.7 vs. 5.9, *p* = 0.002).

Furthermore, one study comprising 80 patients receiving 6SHG or 8SHG reported no differences in IKDC, Lysholm or Tegner scores between single and double‐bundle constructs (*p* = 0.605, 0.438 and 0.262), respectively [23]. Details on subjective clinical measures are summarized in Table 3.

## DISCUSSION

The primary finding of this review was that mean graft diameters for 6SHG and 8SHG ranged from 8.0 to 9.2 mm and 9.1 to 9.9 mm, respectively, exceeding the established 8 mm minimum needed to reduce graft failure rates. The overall graft failure rates and RTS were 4.8% and 75.9%, respectively. Data on PROMs (IKDC, Lysholm and Tegner) demonstrated good patient satisfaction with 6SHG and 8SHG based on previously published criteria. Data on skeletally immature patients were especially lacking; however, the existing evidence suggests that both 6SHG and 8SHG produce sufficient graft diameters in this population.

The importance of graft diameter for ACLR is well‐established in the literature, with several studies reporting a cutoff of 8.0 mm to decrease the risk of graft failure and revision [34, 41]. It has also been reported that for every 0.5 mm increase in graft diameter between 7.0 and 10.0 mm, the revision risk falls by a factor of 0.86 [39]. 4SHG have historically been the most common choice but are often criticized for their unpredictable graft diameters [3]. A recent study reported that 4SHG resulted in a mean diameter of 7.8 mm, with only 24.3% of these grafts exceeding 8 mm in diameter [25]. Thus, hamstring autografts with higher strand numbers have gained popularity as a way to ensure sufficient graft diameters [31, 46]. In this review, all studies that reported on graft diameters of 6SHG had mean values of 8.0 mm or greater, and all studies that reported on graft diameters of 8SHG had mean values of 9.1 mm or greater. This suggests that both constructs reliably produce sufficient graft diameters, which is a crucial advantage over traditional alternatives in terms of graft failure. However, while these findings are positive, there are several concerns regarding using ACLR grafts more strands. In particular, 6SHG and 8SHG are more technically challenging, require prolonged operative time and can compromise graft integrity if performed improperly [31, 47]. Thus, it becomes unclear whether surgeons should always prioritize larger graft diameters despite the risks mentioned above or if there are certain situations in which these risks should take precedence. This uncertainty must be addressed in future studies to better define the indications for higher SHG.

Proper ACLR planning is essential for young, skeletally immature patients as graft failure can have significant consequences, such as accelerated osteoarthritis, increased kinesiophobia and many more [13, 14, 16]. While the superiority of autografts is well‐established [11], the use of BPTB autografts is often discouraged due to the presence of open physes in this population, resulting in hamstring autografts being used instead [31]. Unfortunately, new issues arise since younger age and shorter stature are known predictors of reduced hamstring graft diameter, which is already unpredictable at baseline [28, 44]. This places skeletally immature patients at an especially high risk of graft failure, thus requiring better methods of ensuring sufficient graft diameters, such as increasing strand number. Only two included studies comprising 83 patients reported on paediatric patients receiving 6SHG or 8SHG (mean age 16.0 years) [10, 33], with the mean graft diameters in this subset ranging from 8.5 to 9.2 mm. Given that this range exceeds the established 8.0 mm threshold, these data are encouraging as they indicate the potential for reliable graft sizing in this vulnerable population. Unfortunately, the included studies had no data on paediatric graft lengths. Previous studies have reported that shorter stature in adolescent patients is associated with shorter hamstring grafts [10]. This may prove problematic as sufficient graft length is required for constructs with higher strand numbers due to the increased folds [31]. This may be addressed through alternative surgical techniques, such as the all‐inside technique, but more research is required for definitive conclusions [24]. Furthermore, the physeal status of paediatric patients among the included studies was not specified, which is a crucial distinction that should be made in future studies.

This review found an overall failure rate of 4.8%, while 6SHG and 8SHG‐specific failure rates were 7.1% and 3.9%, respectively. However, one included study with a high graft failure rate of 20% used allograft‐augmented 6SHG, which has been reported to worsen graft survival [33]. Without this study, the 6SHG‐specific failure rate drops to 3.3%, which may represent a more accurate estimation. Previously reported hamstring graft failure rates range from 3% to 12% [16, 19], which suggests that 6SHG and 8SHG produce comparable, if not superior, graft survival compared to other hamstring constructs, possibly owing to a larger graft diameter. Regarding PROMs, pooled averages for the IKDC, Lysholm and Tegner scores were 88.5, 91.9 and 6.9, respectively. Compared to the literature, patient‐acceptable symptom state (PASS) cutoffs for IKDC and Lysholm scores have been reported to be 75.9 and 70.0, respectively; however, no Tegner cutoffs have been reported [6, 29]. This suggests that, at a minimum, both 6SHG and 8SHG constructs result in sufficient patient satisfaction. Furthermore, one included study comprising 38 patients reported that 8SHG constructs resulted in higher IKDC, Lysholm and Tegner scores than 4HS constructs (*p* = 0.007, 0006, 0.002) [49], suggesting potential superiority with higher strand numbers.

This is the first review to summarize the graft dimensions and clinical outcomes of 6SHG and 8SHG constructs for ACLR and highlights the need for more data on this topic. Limitations of this study mainly arose from the limited amount of evidence, with only 13 studies being included. Of these, most included studies constituted Level III/IV evidence, with only four Level I/II studies. As most studies were not prospective, the risk of selection bias is high. The limited number of included patients also reduced the accuracy and precision of the estimated graft dimensions, hence reducing the external validity. Additionally, as mentioned previously, there was a significant lack of data for young, skeletally immature patients despite the literature reporting a higher risk of failure in this population. Hence, this review advocates for considering well‐designed, long‐term prospective cohort studies or RCTs comparing 6SHG and 8SHG with other graft constructs, particularly in skeletally immature patients.

## CONCLUSION

The literature on six and eight‐strand hamstring tendon ACLR demonstrated acceptable graft diameter measurements based on the currently accepted standard of 8.0 mm. Data on PROMs suggested good patient satisfaction based on established criteria of PASS. Re‐rupture and return to sport rates were 4.8% and 75.9%, respectively. Nonetheless, more high‐quality studies are warranted given the current lack of data on the topic, particularly in the paediatric and/or skeletally immature population.

## AUTHOR CONTRIBUTIONS

Bryan Sun contributed to study design, data acquisition, data analysis, data interpretation, manuscript drafting and manuscript revision. Boss Lee and Justin Grad contributed to data acquisition, data analysis and manuscript drafting. Dan Cohen, Jihad Abouali, Sachin Tapasvi and Adit Maniar all critically revised the manuscript. Darren de SA conceived the study, contributed to the study design and revised the manuscript. All authors read and approved the final manuscript to be published and agree to be held accountable for all aspects of the work (i.e., ensuring the accuracy or integrity of any aspect of the work).

## CONFLICT OF INTEREST STATEMENT

The authors declare no conflicts of interest.

## ETHICS STATEMENT

The authors have nothing to report.

## Supporting information

Supporting information.

## Data Availability

All data supporting the findings of this study are available within the article and its Supporting Information. The search strategy used to identify the included studies from online databases is shown in Supporting Information S1: Table 1.
