# Supplementary material for: Anterior cruciate ligament reconstruction with six and eight‐strand hamstring tendon autografts produces adequate graft dimensions and functional outcomes: A systematic review
Source: Knee Surg Sports Traumatol Arthrosc. 2024 Dec 12;33(6):2144–55. doi: 10.1002/ksa.12556 (PMC12104794; doi:10.1002/ksa.12556)
Supplement: Supplementary file 1 — Supporting information. [file KSA-33-2144-s001.docx]

**SUPPLEMENTARY DIGITAL MATERIAL:**

**Supplementary Table 1.** Search Strategy

| 1. anterior cruciate ligament injury/ OR anterior cruciate ligament reconstruction/ OR anterior cruciate ligament/ OR ACL.mp OR ACLR.mp OR reconstruction*.mp |
| --- |
| 2. hamstring tendon/ OR hamstring muscle/ OR hamstring*.mp |
| 3. gracilis.mp |
| 4. semitendinosus muscle/ OR semitendinosus*.mp |
| 5. strand*.mp |
| 6. 2 OR 3 OR 4 |
| 7. 1 AND 5 AND 6 |
